# Golexanolone affords sustained microglia and astrocytes activation improvement in a rat model of Parkinson’s disease

**DOI:** 10.3389/fimmu.2025.1654664

**Published:** 2025-09-03

**Authors:** Gergana Mincheva, Maria A. Pedrosa, Mar Martínez-García Vázquez, Lola Vazquez, Thomas P. Blackburn, Magnus Doverskog, Marta Llansola, Vicente Felipo

**Affiliations:** ^1^ Laboratory of Neurobiology, Centro de Investigación Príncipe Felipe, Valencia, Spain; ^2^ Umecrine Cognition AB, Solna, Sweden

**Keywords:** golexanolone, Parkinson’s disease, neuroinflammation, vimentin, microglia activation, astrocyte activation, striatum, substantia nigra

## Abstract

**Introduction:**

Golexanolone improves motor and non-motor alterations in the unilateral 6-OHDA rat model of PD. We hypothesized that a key mechanism by which golexanolone induces these beneficial effects is by reducing microglia activation, thus reducing pro-inflammatory factors (TNFα, IL-1α, HMGB1) which activate astrocytes. This work aims were to assess if golexanolone affords sustained improvement of glial activation and pro-inflammatory factors at 3 and 9 weeks after 6-OHDA injection.

**Results:**

6-OHDA rats show pro-inflammatory microglia in SN and striatum, with reduced area and increased TNFα at 3 and 9 weeks, increased TNFα, IL-1α and HMGB1 and pro-inflammatory A1 astrocytes activation with increased GFAP, vimentin and S100B and reduced S100A10. Golexanolone reversed microglia activation, the increase in pro-inflammatory factors and astrocytes A1 activation both at 3 and 9 weeks. Golexanolone reversed microglia activation, the increase in pro-inflammatory factors and astrocytes A1 activation both at 3 and 9 weeks.

**Discussion:**

Sustained improvement of glial activation in SN and striatum would be a key mechanism in the improvement of PD symptoms by golexanolone.

## Introduction

Parkinson’s disease (PD) leads to progressive motor and non-motor alterations including fatigue, cognitive impairment, anxiety and depression (1–4). L-DOPA (levodopa), the most usual treatment, improves the main motor symptoms in PD patients but many patients develop L-DOPA induced dyskinesia. Moreover, L-DOPA does not improve cognitive impairment, fatigue, freezing of gait and falls (5, 6). New treatments that improve the L-DOPA resistant motor and non-motor symptoms in PD patients are an unmet need. These new treatments should be directed to improve the pathological mechanisms of PD such as neuroinflammation, glial activation and enhanced GABAergic neurotransmission.

Microglia and astrocytes activation and neuroinflammation play a key role in neuronal loss and in the pathogenesis of most neurodegenerative diseases, including PD (7–9). It has been proposed that targeting microglial or astrocytes activation and neuroinflammation may create new treatment possibilities for PD (10–14).

On the other hand, enhanced GABAergic neurotransmission has also been proposed to play a role in the pathogenesis and symptoms of PD (15–17). Heo et al (16) showed that in substantia nigra (SN) part of the tyrosine hydroxylase (TH) loss is not due to neuronal loss, but to inhibition of TH expression in “dormant” neurons due to enhanced activation of GABA_A_ receptors, suggesting that TH expression and dopamine levels could be partially restored by treatments that reduce GABAergic neurotransmission. Heo et al. (16) showed that inhibiting monoamine oxidase B with safinamide reduces GABA levels, enhances TH expression and improves motor symptoms.

Golexanolone, a GABA_A_ modulating steroid antagonist in clinical development, reduces the activation of GABA_A_ receptors and improves neuroinflammation and cognitive and motor function in rat models of different pathologies (18–22). Golexanolone also improves some motor and non-motor alterations in the unilateral 6-OHDA rat model of PD, without inducing dyskinesia (23).

It has been proposed that there is an interplay between GABAergic neurotransmission and neuroinflammation, which modulate each other (24–27). Treatment with bicuculline, an antagonist of GABA_A_ receptors, reduces some aspects of neuroinflammation in the cerebellum and hippocampus of hyperammonemic rats (28, 29).

**Figure 1:**
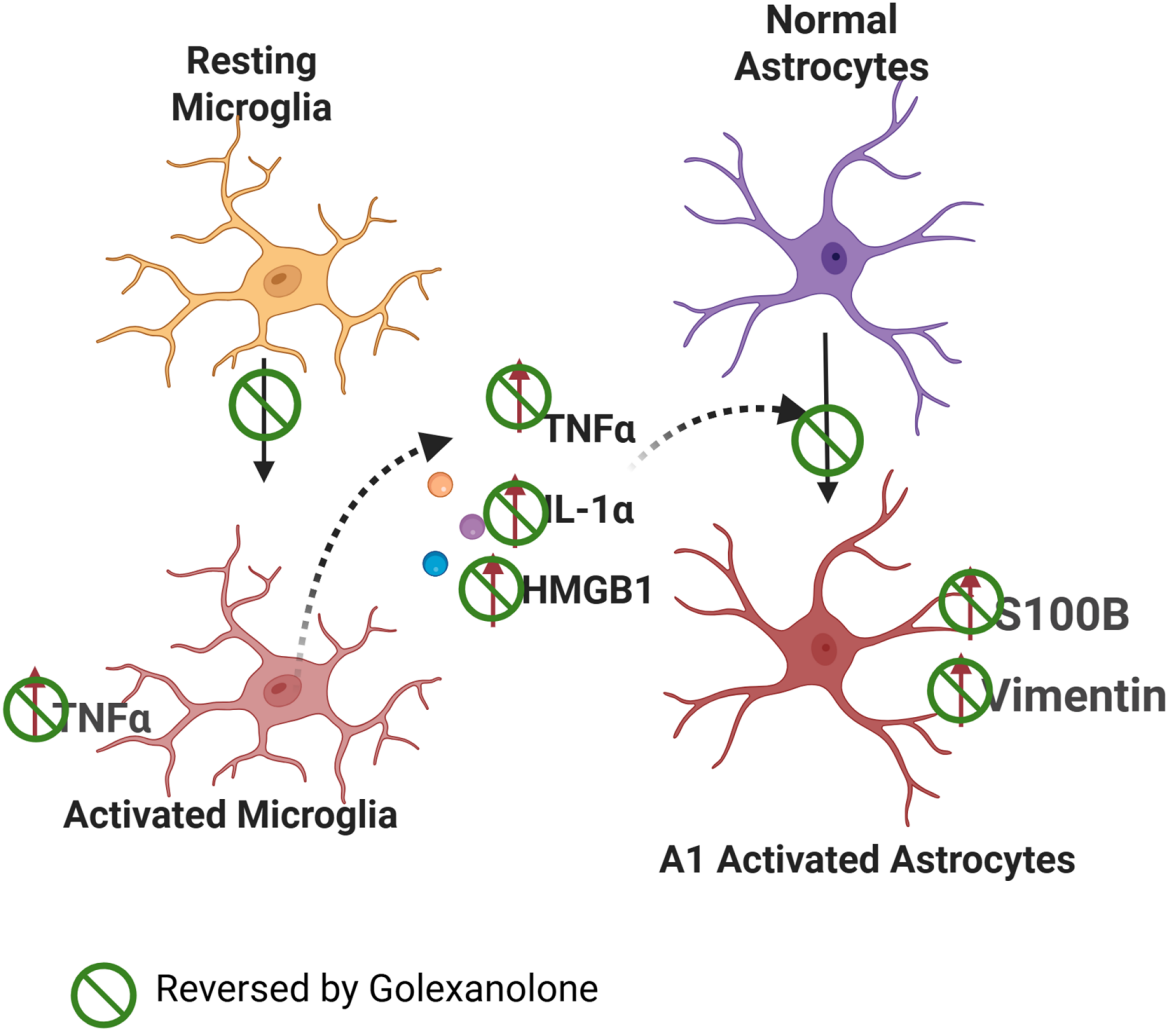
Proposed mechanism of the effects of golexanolone on glial activation. Microglia show a pro-inflammatory activated state in SN and striatum of 6-OHDA rats, with a more ameboid morphology and increased TNFα content. Pro-inflammatory microglia releases TNFα, IL - 1α and HMGB1 which induce activation of astrocytes to an A1 pro-inflammatory form with increased GFAP, vimentin and S100B levels. Golexanolone affords sustained improvement of all the process by reversing microglia activation both at 3 and 9 weeks after 6-OHDA injection. The effects of golexanolone are indicated by 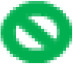.

We therefore hypothesized that a key mechanism by which golexanolone induces beneficial effects in the rat model of PD is by reducing glial activation. In many pathological situations, activated pro-inflammatory microglia releases factors such as TNFa, IL - 1α and HMGB1 that induce activation of astrocytes (**Figure 1**). Reactive A1 pro-inflammatory astrocytes are induced by activated pro-inflammatory microglia, which secrete IL - 1α, TNF α and these cytokines are sufficient to activate astrocytes (30–32). HMGB1 is also released by activated microglia (33).

We also hypothesized that the mechanism by which golexanolone reduces glial activation in the 6-OHDA rat model of PD would involve the process summarized in **Figure 1**, golexanolone reduces pro-inflammatory microglia activation, thus reducing the release of pro-inflammatory factors and astrocytes activation. The first aim of this work was to assess this hypothesis by analyzing the effects of golexanolone on microglia and astrocytes activation and on pro-inflammatory factors in SN and striatum of 6-OHDA rats at 3 weeks after surgery. The second aim was to assess if golexanolone affords sustained improvement of glial activation by analyzing the same parameters in the same brain areas at 9 weeks after surgery.

## Methods

### Rat model of Parkinson’s disease: unilateral 6-OHDA lesion

Eight-week-old Wistar-Han male rats (Charles River, Barcelona) were housed, two per cage, under standard laboratory conditions: 12-hour light–dark cycle, 22 °C room temperature, 55% relative humidity, food and water available *ad libitum*. The experiments were approved by the Comité de Ética y Experimentación Animal (CEEA) of our center and by Conselleria de Agricultura, Generalitat Valenciana, and performed according to the Directive of the European Commission (2010/63/EU) for the care and management of experimental animals and complied with the ARRIVE guidelines for animal research. Unilateral 6-OHDA lesion was performed as in Izquierdo-Altarejos et al (23). Under isoflurane (5% induction and then 2%) anesthesia the animals were placed on a stereotaxic frame with non-traumatic ear bars (Stoelting, USA), and unilaterally injected (right hemisphere) with either vehicle (sham group) or 6-OHDA hydrochloride (Sigma, USA) (6-OHDA group) directly into the medial forebrain bundle (coordinates related to Bregma, AP = −4,4 mm; ML= −1,0 mm; DV = −7,8 mm; according to Paxinos and Watson). At a rate of 1 μl/min, sham rats received 2 μl of 0.2 mg/ml ascorbic acid in 0.9% NaCl and 6-OHDA rats were injected with 2 μl 6-OHDA hydrochloride (4 μg/μl) with 0.2 mg/ml ascorbic acid in 0.9% NaCl. After injection the syringe was left in place for 10 minutes to allow diffusion.

### Apomorphine turning behavior test

To validate the model, the apomorphine-induced turning behavior was evaluated 3 weeks after surgery as in Torres and Dunnett (34). Rats were subcutaneously injected in the neck with 0.1 mg/kg apomorphine hydrochloride (Sigma‐Aldrich) dissolved in 1% of ascorbic acid in 0.9% of NaCl and placed in bowls. 5 min after the injection, rotations were recorded and tracked with Anymaze software during 15 min. The number of net rotations was calculated (contralateral-ipsilateral rotations). Only rats displaying above 4 net rotations/minute were included in the study.

### Treatment

Golexanolone (40 mg/ml in CAPMUL (Capmul^®^ MCM, Glycerol Monocaprylocaprate (Type I) from ABITEC Corporation (Janesville, WI, USA)), or CAPMUL as vehicle, were administered daily using intragastric probes at 50 mg/kg (1.25 ml/kg) as in Mincheva et al (19), starting one week after surgery.

### Experimental design

Rats were divided into the following experimental groups: 1) Sham-operated rats receiving CAPMUL as vehicle (Sham-VH), 2) Sham-operated rats treated with golexanolone (Sham-Golex), 3) 6-OHDA rats receiving CAPMUL as vehicle (6-OHDA-VH) and 4) 6-OHDA rats treated with golexanolone (6-OHDA-Golex). The experimental design included 2 experiments:

Experiment 1: One set of rats (14 Sham-VH, 13 Sham-Golex, 14 6-OHDA-VH and 17 6-OHDA-Golex) was sacrificed three weeks after surgery to perform near-term analyses of glial activation. Golexanolone treatment was administrated for two weeks.

Experiment 2: Another set of rats (15 Sham-VH, 12 Sham-Golex, 15 6-OHDA-VH and 15 6-OHDA-Golex) were sacrificed nine weeks after surgery to perform the analysis of glial activation at a longer time. Golexanolone treatment was administrated for eight weeks.

The total number of animals used in all the study were 115.

### Immunohistochemistry

For immunohistochemistry studies, rats were anesthetized with sodium pentobarbital (300 mg/Kg body weight) and transcardially perfused with 4% paraformaldehyde in 0.1 M phosphate buffer (pH = 7.4). Brains were extracted and post-fixed in the same fixative solution for 24 h. Tissue was embedded in paraffin and 5 µm of coronal sections were cut and mounted on coated slides. Sections were sequentially incubated with 3% H2O2, blocking serum and the following primary antibodies (4°C, overnight): GFAP (1:400, G3893, Sigma) as an astrocyte marker and Iba1 (1:300, 019 - 19741, Wako) as microglial marker. Then, slides were incubated with biotinylated secondary antibodies (1:200, Vector Laboratories) goat anti-mouse or goat anti-rabbit at room temperature for 1h. Signal was amplified using the Vectastain ABC kit (Vector Laboratories). Chromogenic reaction was performed using DAB kit (Abcam) and finally the slides were counterstained with Mayer’s hematoxylin (DAKO) and mounted with DPX. Sections were scanned with an Aperio Versa system (Leica Biosystems, Germany) for further image analysis.

### Analysis of immunohistochemistry

Fields of Striatum and SN at 40x magnification were acquired from the scanned slides using the software ImageScope64. For each rat, 8 – 10 images from each hemisphere (contralateral or ipsilateral) were taken. Microglia activation was analyzed by measuring the perimeter of individual Iba1 stained cells with Image-Pro Plus (v.6.0) software. Astrocytic activation was measured as percentage of GFAP stained area with Image J software.

### Immunofluorescence

Brain coronal sections containing Striatum and SN were deparaffined, hydrated and washed in 0.1 M phosphate buffer. Then they were blocked with donkey serum before being incubated (4°C, overnight) with primary antibodies.

Double immunofluorescences were performed to analyze TNFα (1:150, AF - 510-NA RD, Systems) in Iba1+ cells (Iba1, 1:300, 019 - 19741, Wako) of SN and striatum regions.

For visualization, the sections were incubated with the corresponding combination of Alexa fluor secondary antibodies at a dilution of 1:400: donkey anti-goat-555 (A21432) and donkey anti-rabbit-488 (A32790), Invitrogen. The nuclei were counterstained with DAPI (Sigma-Aldrich) and the sections mounted with Fluoromount-G (Invitrogen).

### Confocal microscopy and immunofluorescence analysis

The sections were observed under a confocal microscope (Leica SP8 HyVOLUTION II) and imaged with a 63x objective lens. Z-stack imaging was obtained from five images per hemisphere (contralateral and ipsilateral) of Striatum and Substantia Nigra from each animal using constant microscope parameters and similar laser intensity. For analysis of TNFα in Iba1+ cells, the cytosol of each Iba1+TNFα positive cell was manually outlined using the freehand selection tool of LasX, and the TNFα mean intensity of each cell was analyzed with ImageJ. The mean of all images of each animal was calculated.

The results on the injured side are expressed as the percentage of the contralateral (un-injured) side to reduce possible antero-posterior differences of the SN (35–37).

### Analysis of protein content by Western blot

Animals were sacrificed by decapitation 3 and 9 weeks after surgery. Striatum and Substantia Nigra were dissected and homogenized in five volumes of lysis buffer (50 mM TRIS–HCl pH 7.5, 50 mM NaCl, 10 mM EGTA, 5 mM EDTA and protease and phosphatase inhibitors) by sonication and after centrifugation at 13000xg for 10 min total protein content was determined in the supernatant by the bicinchoninic acid method (BCA, from PIERCE). Samples (20 µg) were subjected to electrophoresis and immunoblotting as in (38) using the following primary antibodies: TNFα [1:250, AF - 510-NA from RD SISTEMS (Minneapolis, MN, USA)]; HMGB1 [1:1000, AB18256 from ABCAM (Cambridge, UK)]; IL - 1α [1:1000, PA5 – 25921 from Invitrogen (Waltham, MA, USA)]; vimentin [1:5000, GTX100619 from Genetex (Irvine, CA, USA)]; S100B (1:5000 AB52642 from ABCAM) and S100A10 [1:1000 11250 - 1AP from Proteintech (Manchester, UK)].

Membranes were scanned using the Epson Perfection V39 (Epson) and band intensities were quantified using Alpha Imager 2200 version 3.1.3 (Alpha Innotech Corporation). Band intensities were normalized to loading control [GAPDH (1:15000, MAB374, Millipore (Burlington, MA, USA)] or β-actin (1:5000, ab6276, Abcam)) intensity in the same membrane. Results were expressed as percentage of shams.

### Statistical analysis

Data are expressed as mean ± SEM. All statistical analyses were performed using GraphPad Prism software v. 10.2. Data were tested for normality with Kolmogorov–Smirnov or Shapiro-Wilk tests. Statistical analysis was carried out using one‐way ANOVA followed by Tukey’s or Fisher’s LSD multiple comparisons tests or Welch’s ANOVA followed of unpaired t with Welch’s correction test when standard deviations were different between groups. When data did not pass the normality test, the nonparametric Kruskal–Wallis test, with uncorrected Dunn’s test for multiple comparisons, was used. A confidence level of 95% was accepted as significant.

## Results

### Golexanolone improves microglia activation in substantia nigra and striatum at three weeks after 6-OHDA injection

**Figure 2:**
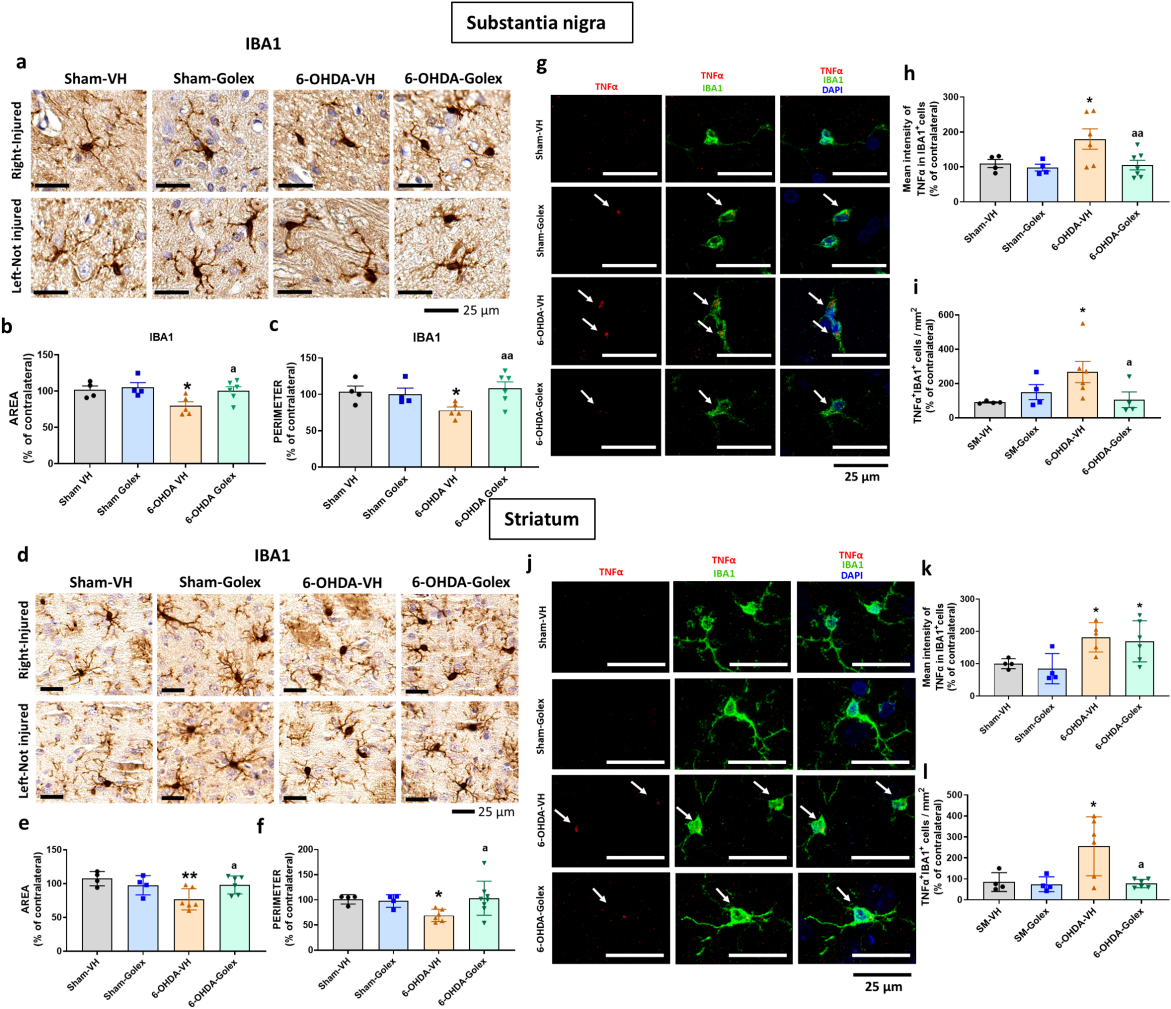
Golexanolone reduces pro-inflammatory microglia in SN and striatum at three weeks after 6-OHDA injection. Representative immunohistochemistry images of Iba1 stained cells in SN **(a)** and striatum **(d)** are shown. Area **(b, e)** and perimeter **(c, f)** of Iba1^+^ cells are represented as mean ± SEM and data were analyzed with one-way ANOVA and uncorrected Fisher’s LSD test in b (F(3,15)=3.805, p=0.033) and e (F(3,16)=4.536, p=0.018) and with Kruskal-Wallis and uncorrected Dunn's test in c (Statistic=7.908, p=0.048) and f (statistic=8.374, p=0.039). Representative confocal microscopy images of TNFα (red) in Iba1^+^ cells (green) by double immunofluorescence of the SN **(g)** and striatum **(j)** in the injured hemisphere. Mean ± SEM of the intensity of TNFα staining into IBA1^+^ cells is shown in SN **(h)** and striatum **(k)**. Data were analyzed with one-way ANOVA and uncorrected Fisher's LSD test: h, F(3,17)=3.857, p=0.028; k, F(3,15)=4.554, p=0.019. The number of Iba1^+^TNFα^+^ cells per area is shown in **(i)**, in SN and in **(l)** in striatum. Data were analyzed with one-way ANOVA and uncorrected Fisher's LSD test in i (F(3,14)=2.831, p=0.077) and with Brown-Forsythe ANOVA followed by unpaired t with Welch's correction in l (F(3.000, 7-088)=7.864, p=0.012). Values significantly different from Sham-VH rats are indicated by asterisks, *p*<0.05, *p<0.01 and values significantly different from 6-OHDA-VH are indicated by a, a p<0.05, aa p<0.01.

Microglia activation is reflected in a reduction of the area and perimeter of microglia cells. 6-OHDA rats show reduced (p<0.05) area (**Figures 2a, b**) and perimeter (**Figures 2a, c**) of microglia cells in SN at 3 weeks after surgery. Golexanolone treatment completely reverses the reduction of area and perimeter (**Figures 2a–c**), indicating that golexanolone reverses microglia activation.

Similar results were obtained in striatum. 6-OHDA rats show a reduced (p<0.05) area (**Figures 2d, e**) and perimeter (**Figures 2d, f**) of microglia cells in striatum at 3 weeks after surgery. Golexanolone treatment completely reverses the reduction of area and perimeter (**Figures 2d–f**), indicating reversal of microglia activation.

Pro-inflammatory microglia show increased levels of TNFα. We also analyzed the levels of TNFα in microglia by double immunofluorescence with Iba1 and TNFα. 6-OHDA rats show increased pro-inflammatory microglia in SN at 3 weeks after surgery, as indicated by the increased content of TNFα (**Figures 2g, h**) in microglia cells and the increased number of TNFα- positive microglial cells (**Figures 2g, i**). Golexanolone treatment completely reverses the increase in TNFα intensity and in TNFα-positive microglial cells (**Figures 2g–i**), indicating reversal of pro-inflammatory activation of microglia.

Similar results were observed in striatum, 6-OHDA rats show increased pro-inflammatory microglia in striatum at 3 weeks after surgery, as indicated by the increased content of TNFα (**Figures 2j, k**) in microglia cells and the increased number of TNFα-positive microglial cells (**Figures 2j, l**). Golexanolone treatment does not reverse the increase in the intensity of the TNFα staining per cell, but strongly reduced the number of pro-inflammatory TNFα-positive microglial cells (**Figures 2j, l**), indicating reversal of pro-inflammatory activation of microglia.

### Golexanolone treatment reduces inflammatory factors released by activated microglia differentially in SN and striatum, at three weeks after 6-OHDA injection

Activated pro-inflammatory microglia may release inflammatory factors which, in turn activate astrocytes. These factors include TNFα, IL - 1α and HMGB1. We therefore analyzed the content of these factors by Western blot.

**Figure 3:**
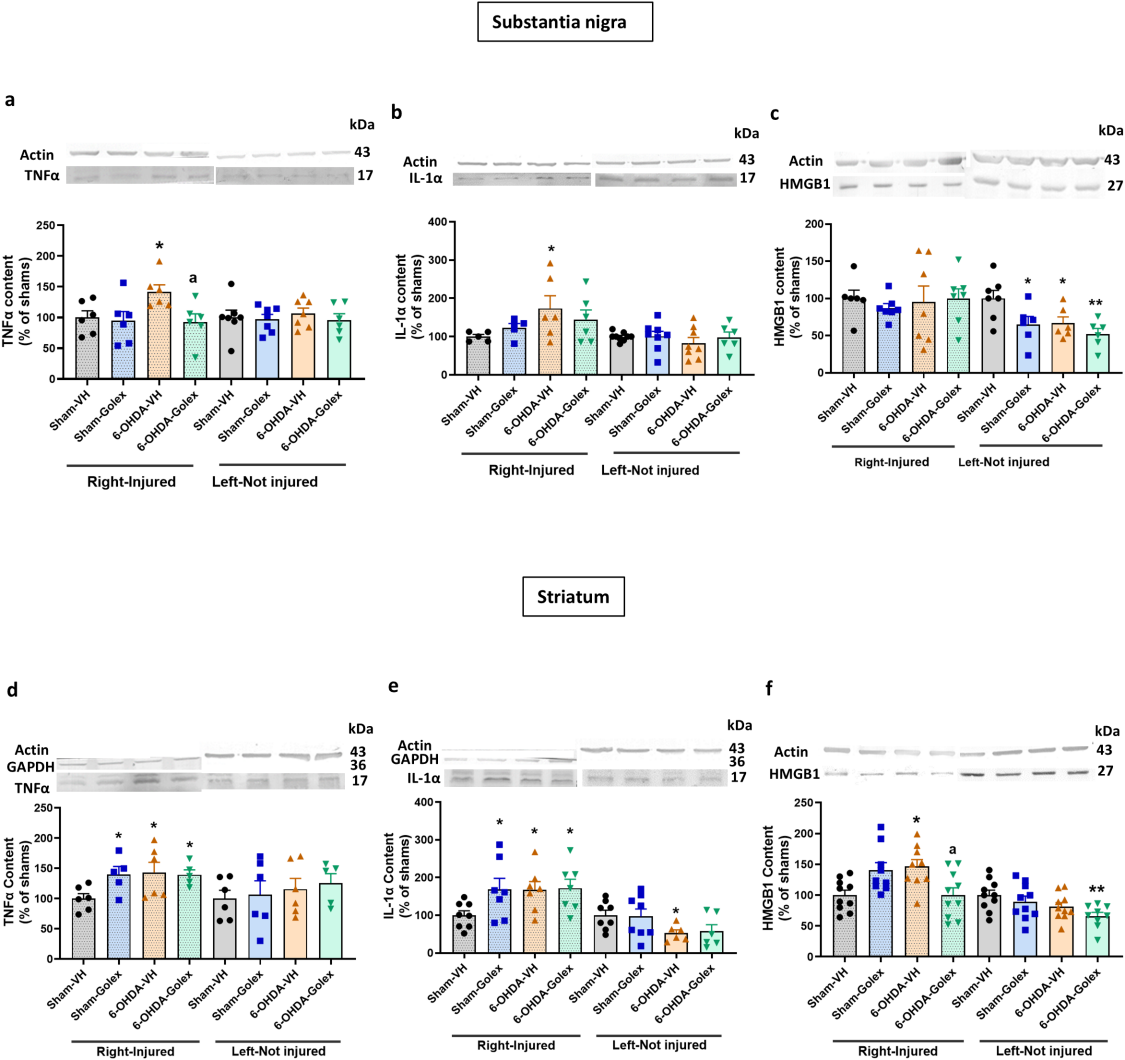
Golexanolone reverses the increase of the pro-inflammatory factors TNFα and IL - 1α in SN and of HMGB1 in the striatum at three weeks after 6-OHDA injection. The content of the pro-inflammatory factors TNFα, IL - 1α and HMGB1 in SN and striatum was analyzed by Western blot and are expressed as mean ± SEM of percentage of sham-vehicle group. **(a)** TNFα in SN. Data were analyzed with one-way ANOVA and uncorrected Fisher’s LSD test, independently in the right and left hemisphere: F(3,20)=3.305, p=0.041 for right and F(3,23)=0.233. p=0.87 for the left. **(b)** IL - 1α in SN. Data were analyzed with one-way ANOVA and uncorrected Fisher’s LSD test for right: F(3,18)=1.746, p=0.19 and left: F(3,27)=0.5200, p=0.67. **(c)** HMGB1 in SN. Data were analyzed with Welch’s ANOVA and unpaired t with Welch’s correction test for the right hemisphere: W(3.000, 11.56)=0.4673, p=0.71 and with one-way ANOVA and uncorrected Fisher’s LSD test for the left: F(3,21)=4.735, p=0.011. **(d)** TNFα in striatum. Data were analyzed with one-way ANOVA and uncorrected Fisher’s LSD test for right: F(3,18)=2.756, p=0.073 and left: F(3,19)=0.3614. **(e)** IL - 1α in striatum. Data were analyzed with one-way ANOVA and uncorrected Fisher’s LSD test for right: F(3,25)=2.599, p=0.075 and with Welch’s ANOVA and unpaired t with Welch’s correction test for the left: W(3.000,12.68)=3.463, p=0.049. **(f)** HMGB1 in striatum. Data were analyzed with one-way ANOVA and uncorrected Fisher’s LSD test for right: F(3,34)=5.185, p=0.0047 and left: F(3,35)=3.431, p=0.027. Values significantly different from Sham-VH rats are indicated by asterisks, *p<0.05, **p<0.01 and values significantly different from 6-OHDA-VH are indicated by a, a p<0.05.

At 3 weeks after surgery, 6-OHDA rats show increased (p<0.05) levels of TNFα (**Figure 3a**) and IL - 1α (**Figure 3b**) in the injured side of SN while HMGB1 was not significantly altered (**Figure 3c**). Golexanolone treatment completely reversed the increases in TNFα (**Figure 3a**) and IL - 1α (**Figure 3b**) levels.

In striatum 6-OHDA rats show increased (p<0.05) levels of TNFα (**Figure 3d**), IL - 1α (**Figure 3e**) and HMGB1 (**Figure 3f**). In striatum, golexanolone treatment reversed the increase in HMGB1 (**Figure 3f**) but not those of TNFα (**Figure 3d**) and IL - 1α (**Figure 3e**).

### Golexanolone reduces astrocytes activation in substantia nigra and striatum at three weeks after 6-OHDA injection

The release by microglia of TNFα, IL - 1α and HMGB1 induces activation of astrocytes to the A1 pro-inflammatory form. Astrocytes pro-inflammatory activation is reflected in increased area stained by anti-GFAP, increased levels of vimentin and S100B while the A2 anti-inflammatory form is associated with increased levels S100A10 (31, 32, 39–41).

**Figure 4:**
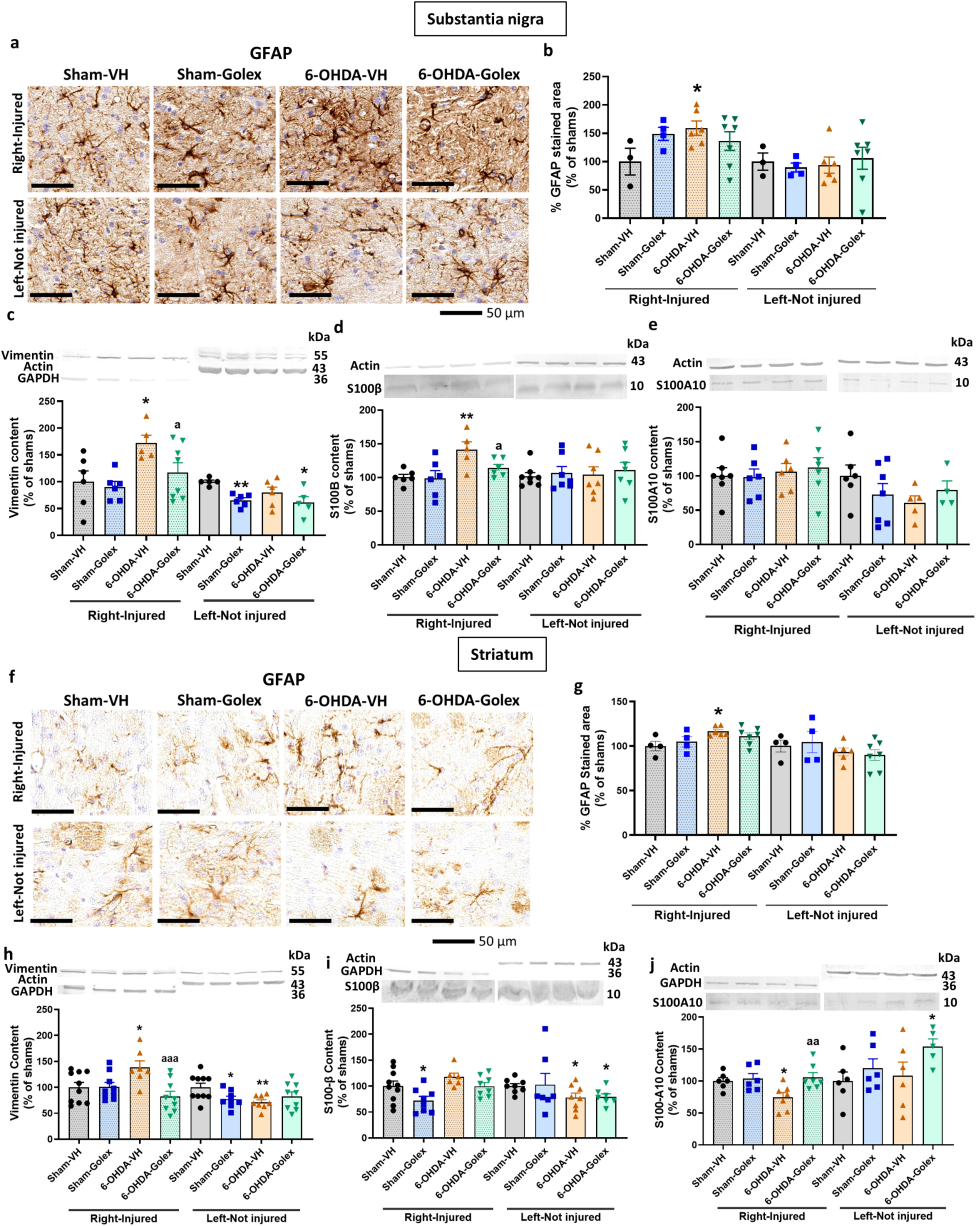
Golexanolone reduces astrocyte activation and the increase of activation markers vimentin and S100B in SN and striatum at three weeks after 6-OHDA injection. Representative immunohistochemistry images of GFAP stained astrocytes are shown in **(a)** for SN and in **(f)** for the striatum. The mean ± SEM of the area stained by GFAP is given as percentage of sham-vehicle rats in **(b)** for SN and in **(g)** for the striatum. Data were analyzed with one-way ANOVA followed by uncorrected Fisher’s LSD test, independently in the right and left hemisphere: F(3,16)=1.885, p=0.17 for the right and F(3,16)=0.1837, p=0.91 for the left in SN **(b)**; F(3,17)=2.907, p=0.065 for the right and F(3,17)=0.8376, p=0.49 for the left in the striatum **(g)**. The markers of activated astrocytes vimentin, S100B and S100A10 were analyzed by western blot in SN and striatum and the mean ± SEM of percentage of sham-vehicle group is shown. Data were analyzed with one-way ANOVA followed by uncorrected Fisher’s LSD test, independently in the right and left hemisphere. **(c)** Vimentin in SN: F(3,21)=3.909, p=0.023 for the right and F(3,18)=4.435, p=0.017 for the left. **(d)** S100B in SN: F(3,19)=4.764, p=0.012 for right and F(3,25)=0.1784, p=0.91 for the left. **(e)** S100A10 in SN: F(3,22)=0.2478, p=0.86 for the right and F(3,18)=0.210, p=0.33 for the left. **(h)** Vimentin in striatum: F(3,31)=4.966, p=0.0063 for the right and F(3,31)=3.141, p=0.039 for the left. **(i)** S100B in striatum: F(3,28)=4.173, p=0.015 for the right and W(3.000,13.38)=3.102, p=0.063 for the left. **(j)** S100A10 in striatum: F(3,22)=4.326, p=0.015 for the right and F(3,19)=1.939, p=0.16 for the left. Values significantly different from Sham-VH rats are indicated by asterisks, *p<0.05, **p<0.01 and values significantly different from 6-OHDA-VH are indicated by a, a p<0.05, aa p<0.01, aaa p<0.001.

At 3 weeks after surgery, 6-OHDA rats show increased area stained by anti-GFAP in SN (**Figures 4a, b**), increased levels of vimentin (**Figure 4c**) and of S100B (**Figure 4d**) in the injured side with no change in S100A10 (**Figure 4e**). This indicates the activation of astrocytes in SN. Treatment with golexanolone reversed activation of astrocytes, as reflected in the complete reversal of the increase in GFAP staining (**Figures 4a, b**) and of the levels of vimentin (**Figure 4c**) and of S100B (**Figure 4d**).

6-OHDA rats also show astrocytes activation in striatum, as indicated by the increased area stained by anti-GFAP (**Figures 4f, g**), increased levels of vimentin (**Figure 4h**) and reduced levels of S100A10 (**Figure 4j**) in the injured side with no change in S100B (**Figure 4i**). Treatment with golexanolone reversed activation of astrocytes, as reflected in the reversal of the increase in GFAP staining (**Figures 4f, g**) and of the levels of vimentin (**Figure 4h**) and of the reduction in S100A10 (**Figure 4j**).

The above results show that golexanolone treatment affords a robust protection against microglia and astrocytes activation at 3 weeks after surgery. To assess if golexanolone affords sustained protection for long periods of time, we analyzed the same parameters at 9 weeks after surgery.

### Golexanolone also reduces pro-inflammatory microglia in SN and striatum at nine weeks after surgery

**Figure 5:**
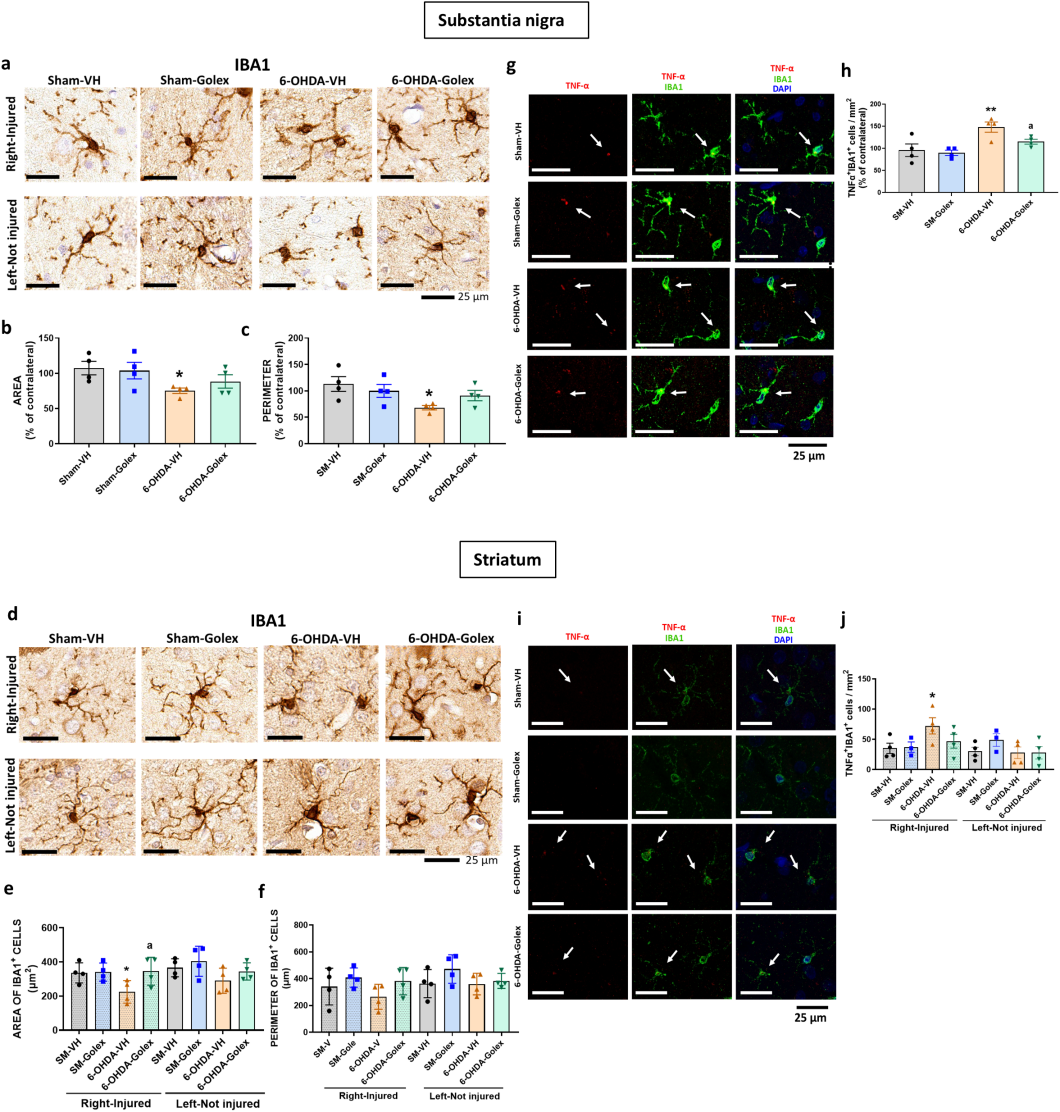
Golexanolone reduces pro-inflammatory microglia in SN and striatum nine weeks after 6-OHDA injection. Representative immunohistochemistry images of Iba1 stained cells in SN **(a)** and striatum **(d)** are shown. Perimeter **(c, f)** and area **(b, e)** of Iba1^+^ cells are represented as mean ± SEM and data were analyzed with one-way ANOVA and uncorrected Fisher’s LSD test: b) (F(3,12)=2.623, p=0.099), e) (F(3,12)=3.089, p=0.068 for right hemisphere and F(3,12)=1.964, p=0.17 for the left, c) F(3,12)= 3.097, p=0.067 and f) (3,12)=1.449, p=0.28 for right and F(3,12)=1.381, p=0.30 for left. Representative confocal microscopy images of TNFα (red) in Iba1^+^ cells (green) by double immunofluorescence of the SN **(g)** and striatum **(i)** in the injured hemisphere. Mean ± SEM of the number of Iba1^+^TNFα^+^ cells per area, as percentage of the contralateral hemisphere in SN is shown in **(h)**. Data were analyzed by one-way ANOVA (F(3,12)=6.861, p=0.006. In the striatum the mean± SEM of the number of Iba1^+^TNFα^+^ cells per area is shown in both hemispheres **(j)**. Data were analyzed by one-way ANOVA: (F(3,11)=2.45, p=0.12 in the right hemisphere and F(3,11)=1.032, p=0.42 in the left. Values significantly different from Sham-VH rats are indicated by asterisks, *p*<0.05, *p<0.01 and values significantly different from 6-OHDA-VH are indicated by a, a p<0.05.

At 9 weeks after surgery, 6-OHDA rats show reduced (p<0.05) area (**Figures 5a, b**) and perimeter (**Figures 5a, c**) of microglia cells in SN. Golexanolone treatment partially reverses the reduction of area and perimeter (**Figures 5a–c**).

In striatum, 6-OHDA rats show reduced area (**Figures 5d, e**) and a tendency to reduce the perimeter (**Figures 5d, f**) of microglia cells at 9 weeks after surgery. Golexanolone treatment completely reverses the reduction of area and perimeter (**Figures 5d–f**), indicating improvement of microglia activation.

6-OHDA rats also show increased pro-inflammatory microglia in SN at 9 weeks after surgery, as indicated by the increased number of TNFα- positive microglial cells (**Figures 5g, h**). Golexanolone treatment completely reverses the increase in TNFα-positive microglial cells (**Figures 5g, h**), indicating reversal of pro-inflammatory activation of microglia.

Similar results were observed in striatum, 6-OHDA show increased pro-inflammatory microglia at 9 weeks after surgery, as indicated by the increased number of TNFα-positive microglial cells (**Figures 5i, j**). Golexanolone completely reverses the increase in the number of pro-inflammatory TNFα-positive microglial cells (**Figures 5i, j**), indicating reversal of pro-inflammatory activation of microglia.

### Golexanolone treatment reduces inflammatory factors released by activated microglia in SN and striatum at nine weeks after 6-OHDA injection

**Figure 6:**
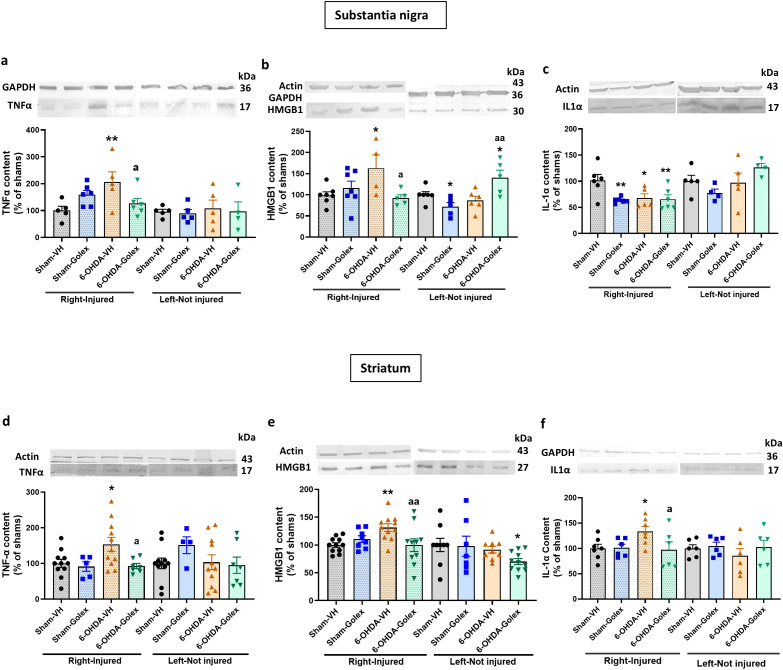
Golexanolone reverses the increase of pro-inflammatory factors in SN and striatum at nine weeks after 6-OHDA injection. The content of the pro-inflammatory factors TNFα, IL - 1α and HMGB1 in SN and striatum was analyzed by Western blot and are expressed as mean ± SEM of percentage of sham-vehicle group. Data were analyzed by one-way ANOVA and uncorrected Fisher’s LSD test, independently in the right and left hemisphere **(a)** TNFα in SN: F(3,18)=3.577, p=0.035 for right and F(3,15)=0.1025. p=0.96 for the left. **(b)** HMGB1 in SN: F(3,19)=3.202, p=0.047 for the right hemisphere and F(3,18)=6.191, p=0.0045 for the left. **(c)** IL - 1α in SN: F(3,19)=4.712, p=0.013 for right and F(3,14)=2.183, p=0.1355 for left. **(d)** TNFα in striatum: for the right hemisphere Welch’s ANOVA test was used (F*(3.000,22.28)=5.155, p=0.0074) and F(3,29)=0.9063, p=0.45 for the left hemisphere. **(e)** HMGB1 in striatum: F(3,36)=4.136, p=0.013 in the right and F(3,32)=2.004, p=0.13 in the left. **(f)** IL - 1α in striatum: F(3,22)=2.805, p=0.064 for right and F(3,20)=0.6438, p=0.60 for left. Values significantly different from Sham-VH rats are indicated by asterisks, *p<0.05, **p<0.01 and values significantly different from 6-OHDA-VH are indicated by a, a p<0.05, aa p<0.01.

At 9 weeks after surgery, 6-OHDA rats show increased levels of TNFα (**Figure 6a**) and HMGB1 (**Figure 6b**) while IL - 1α levels are reduced (**Figure 6c**) in the injured side of SN. Golexanolone treatment completely reversed the increases in TNFα (**Figure 6a**) and HMGB1 (**Figure 6b**) levels and did not affect IL - 1α levels (**Figure 6c**).

In striatum 6-OHDA rats show increased levels of TNFα (**Figure 6d**), HMGB1 (**Figure 6e**) and IL - 1α (**Figure 6f**). Golexanolone treatment completely reversed the increases in TNFα (**Figure 6d**), HMGB1 (**Figure 6e**) and IL - 1α (**Figure 6f**).

### Golexanolone reduces astrocyte activation in substantia nigra at nine weeks after 6-OHDA injection

**Figure 7:**
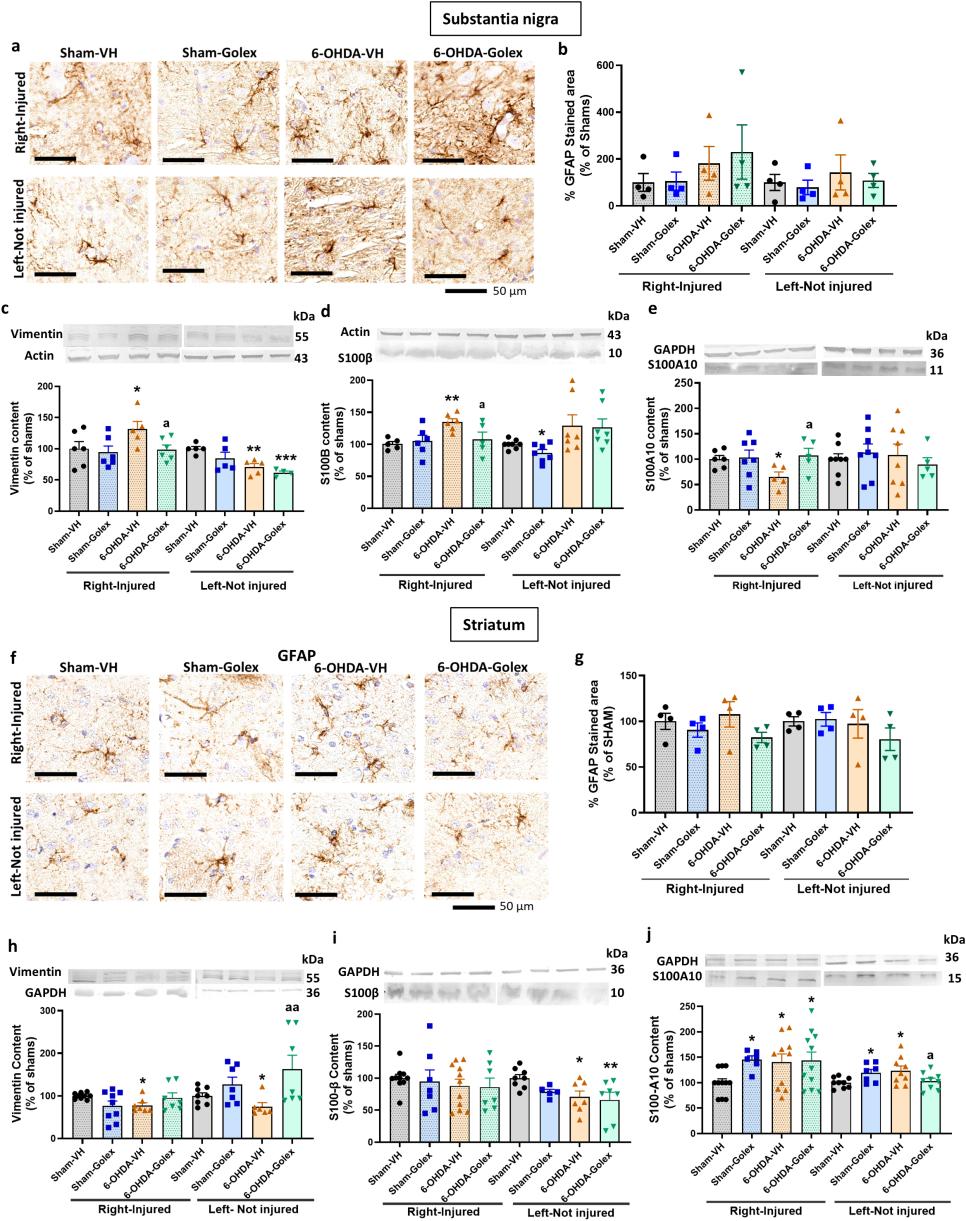
Golexanolone improves astrocyte activation in SN nine weeks after 6-OHDA injection. Representative immunohistochemistry images of GFAP stained astrocytes are shown in **(a)** for SN and in **(f)** for the striatum. The mean ± SEM of the area stained by GFAP is given as percentage of sham-vehicle rats in **(b)** for SN and in **(g)** for the striatum. Data were analyzed with Kruskal-Wallis test followed by uncorrected Dunn’s test in SN **(b)**, independently in the right and left hemisphere: statistic=3.044, p=0.41 for the right and statistic=0.7279, p=0.89 for the left. In the striatum **(g)**, data were analyzed by one-way ANOVA and uncorrected Fisher’s LSD test: F(3,10)=5.19, p=0.02 for the right and F(3,10)=2.155, p=0.16 for the left. The markers of activated astrocytes vimentin, S100B and S100A10 were analyzed by western blot in SN and striatum and the mean ± SEM of percentage of sham-vehicle group is shown. Data were analyzed with one-way ANOVA followed by uncorrected Fisher’s LSD test, independently in the right and left hemisphere. **(c)** Vimentin in SN: F(3,19)=2.519, p=0.089 for the right and F(3,15)=6.784, p=0.0041 for the left. **(d)** S100B in SN: F(3,18)=4.009, p=0.024 for right and Kruskal-Wallis test and uncorrected Dunn’s test were used for the left: statistic=11.81, p=0.0081. **(e)** S100A10 in SN: F(3,19)=2.257, p=0.11 for the right and F(3,25)=0.3294, p=0.80 for the left. **(h)** Vimentin in striatum: Welch’s ANOVA test was used, followed by unpaired t with Welch’s correction (W(3.000,12.30)=4.241, p=0.16) for the right and Kruskal-Wallis test and uncorrected Dunn’s test were used for the left: statistic=9.203, p=0.027. **(i)** S100B in striatum: F(3,31)=0.3071, p=0.82 for the right and F(3,23)=3.394, p=0.035 for the left. **(j)** S100A10 in striatum: Welch’s ANOVA test was used, followed by unpaired t with Welch’s correction (W(3.000,17.90)=6.011, p=0.051) for the right and F(3,30)=3.307, p=0.033 for the left. Values significantly different from Sham-VH rats are indicated by asterisks, *p<0.05, **p<0.01. *** p<0.001 and values significantly different from 6-OHDA-VH are indicated by a, a p<0.05, aa p<0.01.

At 9 weeks after surgery, the area stained by anti-GFAP tended to be higher but was not significantly different in 6-OHDA rats than in control rats in SN (**Figures 7a, b**). However, astrocytes are activated in 6-OHDA rats, as indicated by the increased levels of vimentin (**Figure 7c**) and of S100B (**Figure 7d**) and the reduced levels of S100A10 (**Figure 7e**) in the injured side. Treatment with golexanolone reversed activation of astrocytes, as reflected in the complete reversal of the increase in the levels of vimentin (**Figure 7c**) and of S100B (**Figure 7d**) and of the reduction of S100A10 (**Figure 7e**) in SN.

6-OHDA rats do not show astrocytes activation in striatum, as indicated by the lack of differences in the area stained by anti-GFAP (**Figures 7f, g**). Moreover, in striatum at 9 weeks after surgery we did not observe increased but reduced levels of vimentin (**Figure 7h**) and increased levels of S100A10 (**Figure 7j**) in the injured side with no change in S100B (**Figure 7i**). Treatment with golexanolone normalized the levels of vimentin (**Figure 7h**) and did not affect the other parameters (**Figure 7**).

## Discussion

The results reported are summarized in **Table 1** and **Figure 1**. In **Table 1** the effects reversed by golexanolone are highlighted with a green background. In aggregate, golexanolone reverses nearly all the harmful effects of the 6-OHDA model on glial activation.

**Table 1:** Summary of the effects of 6-OHDA injection and of golexanolone on different parameters in SN and striatum at 3 and 9 weeks after 6-OHDA injection.

Time after 6-OHDA	3 weeks		9 weeks	
Brain area	SN	Striatum	SN	Striatum
Microglia activation				
Iba1 IHQ	**Increased**	**Increased**	**Increased**	**Increased**
TNFα in Iba1+	**Increased**	**Increased**	**Increased**	**Increased**
Pro-inflammatory factors				
TNFα	**Increased**	**Increased**	**Increased**	**Increased**
IL-1α	**Increased**	**Increased**	**Reduced**	**Increased**
HMGB1	**Not affected**	**Increased**	**Increased**	**Increased**
Astrocytes activation				
GFAP IHQ	**Increased**	**Increased**	**Not affected**	**Not affected**
S100B	**Increased**	**Not affected**	**Increased**	**Not affected**
Vimentin	**Increased**	**Increased**	**Increased**	**Reduced**
S100A10	**Not affected**	**Reduced**	**Reduced**	**Increased**
A1 astrocytes	**Increased**	**Increased**	**Increased**	**Reduced**
A2 astrocytes	**Not affected**	**Reduced**	**Reduced**	**Increased**

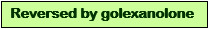

See text for details.

The reason for say that golexanolone reverse instead of avoid is because microglia activation is a very fast response to injection of 6-OHDA (42). We show here that microglia is activated in 6-OHDA rats 3 weeks after surgery. Moreover, we have previously shown that starting the golexanolone treatment at 4 weeks after surgery, after microglia activation, reverses the activation of microglia in striatum (23). This clearly shows that golexanolone reverses the activation of microglia.

6-OHDA rats show pro-inflammatory microglia in SN and striatum, with reduced area and perimeter and increased TNFα levels both at 3 and 9 weeks after surgery. It has been shown that activated microglia releases pro-inflammatory factors such as TNFα, IL - 1α and HMGB1 which trigger pro-inflammatory A1 activation of astrocytes (30–32). We show here that 6-OHDA rats also show increased levels of these factors in SN and striatum at 3 and 9 weeks after surgery. These factors would induce pro-inflammatory A1 astrocytes, as indicated by the increased GFAP immunostaining and the increased content of S100B and vimentin. Neuroprotective A2 astrocytes are characterized by the expression of S100A10 (32). We also show that the content of S100A10 is reduced in striatum at 3 weeks and in SN at 9 weeks after surgery, indicating a reduction of neuroprotective A2 astrocytes.

All these changes are reversed by golexanolone treatment, which reduces pro-inflammatory activation of microglia and astrocytes and the levels of pro-inflammatory factors in SN and striatum both at 3 and 9 weeks after surgery. A noteworthy beneficial effect of golexanolone is the reversal of microglia activation. Golexanolone affords a complete and sustained improvement of microglia activation in SN and striatum reducing pro-inflammatory microglia as reflected by the normalization of area, perimeter and TNFα content. We also analyzed another proinflammatory cytokine that would be involved in the inflammatory process. Content of IL - 1β was increased in the striatum, only at long time, but this increase was not affected by golexanolone, indicating that the increase of this cytokine is not mediated by pro-inflammatory microglia and is not involved in the anti-inflammatory effect of golexanolone (data not shown).

In the striatum of sham rats after two weeks of goloxanolone administration, we observed increased levels of TNFα, IL - 1α, and HMGB1 (although not significant in this case). This indicates an increase in proinflammatory factors, although microglial morphology and TNFα expression in microglia are not altered. Astrocytic activation was also not observed in this experimental group at this time point. This suggests that other cells, possibly neurons with GABA_A_ receptors, could be transiently increasing the levels of these proinflammatory proteins in response to goloxanolone, as this is no longer observed after prolonged treatment with goloxanolone (8 weeks). Early and transient increased expression of these proteins by golexanolone would not be sufficient to induce glial activation, as microglia or astrocyte activation is not found in striatum in this group. This explanation is supported by reports showing neuronal expression of HMGB1 and association of HMGB1 with GABA signaling (43–46) as well as modulation of HMGB1 levels in brain by neurosteroids (47).

We observe some differences in the astrocyte responses between SN and striatum in 6-OHDA rats. In SN the content of S100B, a marker of A1 pro-inflammatory astrocytes, is increased in 6-OHDA rats both at 3 and 9 weeks after surgery. However, in striatum of the same rats S100B is not altered. A similar effect has been reported by Monnot et al (48) using the BSSG rat model of PD. They showed that in these rats S100B is increased in SN but not in striatum and that the increase of S100B was observed at long but not at short times after BSSG treatment. Similar differences have also been reported in senescence, which reduces S100B in SN but not in striatum (49). Concerning the changes in the 6-OHDA model there are conflicting reports. Batassini et al (50) did not find any increase of S100B in SN or striatum at 7 or 21 days after 6-OHDA injection. Gordon et al (51) found increased levels of GFAP but not of S100B in striatum. However, Morales et al (52) reported increased S100B and vimentin in striatum 5 days after 6-OHDA injection. Sathe et al (13) found increased levels of S100B in SN of patients with PD and of mice treated with MPTP, another model of PD.

Here we found an increase in S100B in SN at 3 and 9 weeks after surgery which are completely reversed by golexanolone, indicating improvement of pro-inflammatory astrocytes, as further confirmed by the reduction of GFAP staining and vimentin content.

The alterations in astrocytes progress with time in the 6-OHDA rats both in SN and, especially, in striatum. In SN pro-inflammatory A1 astrocytes are increased at 3 and 9 weeks as indicated by increased GFAP staining and vimentin and S100B content. Neuroprotective A2 astrocytes are not affected at 3 weeks but are reduced at 9 weeks, as shown by reduced S100A10 content. This would reflect an increased harmful astrocytic response at 9 weeks compared to 3 weeks. Golexanolone completely reversed all harmful changes in astrocytes in SN at both 3 and 9 weeks after surgery.

Concerning the striatum, pro-inflammatory A1 astrocytes are increased and neuroprotective A2 astrocytes reduced at 3 weeks (see **Table 1**), indicating a more harmful astrocytic response in striatum than in SN at early stages. Golexanolone completely prevents this harmful response. In contrast, at 9 weeks, pro-inflammatory A1 astrocytes are reduced in striatum, with no changes in GFAP staining and reduced vimentin content, while A2 neuroprotective astrocytes are increased, as reflected by the increase in S100A10. This suggests an adaptive response to prevent the damage generated by pro-inflammatory astrocytes. These data agree with our previous study showing increased astrocytes activation at 5 but not at 10 weeks after 6-OHDA injection (23). Golexanolone prevents the reduction of vimentin and does not affect the increase of A2 neuroprotective astrocytes. In fact, golexanolone increases A2 astrocytes (S100A10) in control rats at 9 weeks.

These data clearly show that golexanolone affords a sustained improvement of glial activation in SN and striatum of 6-OHDA rats. Golexanolone treatment improves motor incoordination, certain aspects of locomotor gait, fatigue, anxiety, depression, and short-term memory in 6-OHDA rats (23). Sustained improvement of glial activation in SN and striatum would be a key mechanism in the improvement of PD symptoms by golexanolone.

Neuroinflammation plays a key role in the pathogenesis of PD. Neuroinflammation and microglia and astrocytes activation have been proposed as therapeutic targets for the treatment of PD (53–59). PD patients show A1 reactive astrocytes in SN, as indicated by the increased immunostaining of S100B (13). These authors propose a role of S100B in the pathophysiology of PD and that targeting S100B may emerge as a potential treatment strategy in PD. A1 pro-inflammatory astrocytes, with increased S100B levels, are also present in SN in a rat model of PD induced by intranigral administration of BSSG (14). These authors propose that activated microglia release factors that trigger pro-inflammatory A1 astrocytes activation and that new therapies should aim to block the conversion of A1 astrocytes by activated microglia (14).

Reactive A1 pro-inflammatory astrocytes are induced by activated pro-inflammatory microglia, which secrete IL - 1α, TNF α and these cytokines are sufficient to activate astrocytes (30–33).

Taken together, these reports suggest that a good procedure to prevent pro-inflammatory A1 astrocytes activation would be to reduce pro-inflammatory microglia activation. The results reported here support this idea. Reducing pro-inflammatory microglia activation with golexanolone is associated with reduced pro-inflammatory activation of astrocytes which would contribute to the improvement of PD symptoms as suggested by Sathe et al (13), Rocha et al (53), Rappold and Tieu (56), Miyazaki and Asanuma (57) and Luna-Herrera et al (14).

Moreover, improving microglia activation affords additional benefits including the reduction of pro-inflammatory factors and the effects of these factors on neurons and has also been proposed as a therapeutic target in Parkinson’s and neurodegenerative diseases (9, 58, 59).

It has also been proposed that there is an interplay between GABAergic neurotransmission and neuroinflammation, which modulate each other (24–27). Although the reports on the effects of GABA on microglia activation are controversial, some reports suggest that GABA enhances microglia activation (60). Treatment with bicuculline, an antagonist of GABA_A_ receptors, reduces pro-inflammatory microglia activation in the cerebellum of hyperammonemic rats (27), a model of peripheral- and neuroinflammation.

Similarly, being a novel GABA_A_ modulating steroid antagonist, the results reported here therefore show that golexanolone reduces pro-inflammatory microglia activation, pro-inflammatory factors and subsequent activation of astrocytes (**Figure 1**).

These results also suggest that golexanolone would also reduce glial activation and neuroinflammation in other neurodegenerative diseases involving neuroinflammation, and therefore its therapeutic use could be extended to these diseases. This suggests that future research on the effect of golexanolone in preclinical models of these neuroinflammatory diseases, such as Alzheimer’s or Huntington’s disease, is warranted.

## Conclusions

6-OHDA rats show pro-inflammatory microglia in SN and striatum, with reduced area and perimeter and increased TNFα levels both at 3 and 9 weeks after surgery. This is associated with increased levels of pro-inflammatory factors TNFα, IL - 1α and HMGB1 and pro-inflammatory A1 astrocytes activation as indicated by increased GFAP immunostaining and levels of vimentin and S100B and reduced levels of S100A10. Golexanolone treatment reversed microglia activation, the increase in pro-inflammatory factors and astrocytes A1 activation both at 3 and 9 weeks after surgery. Sustained improvement of glial activation in SN and striatum would be a key mechanism in the improvement of PD symptoms by golexanolone, adding to the validation of this group of neurosteroid-based compounds to treat neurological symptoms caused by neuroinflammation and aberrant GABAergic neurotransmission.

## Data Availability

The raw data supporting the conclusions of this article will be made available by the authors, without undue reservation.
